# Supplementary material for: Molecular mapping and functional validation of GLP-1R cholesterol binding sites in pancreatic beta cells
Source: eLife. 2025 Apr 24;13:RP101011. doi: 10.7554/eLife.101011 (PMC12021413; doi:10.7554/eLife.101011)
Supplement: Figure 3—figure supplement 1—source data 1. [file elife-101011-fig3-figsupp1-data1.pdf]

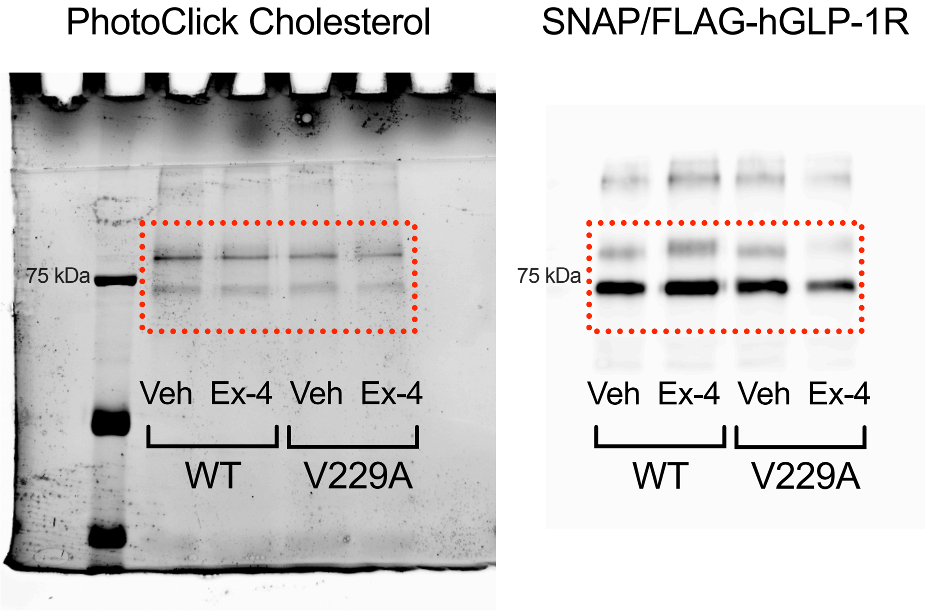

Figure 3-figure supplement 1-Source data 1. PDF file containing original western blots for Figure 3-figure supplement 1A, indicating the relevant bands and treatments.
