# Supplementary figures and images for: Molecular mapping and functional validation of GLP-1R cholesterol binding sites in pancreatic beta cells
Source: eLife. 2025 Apr 24;13:RP101011. doi: 10.7554/eLife.101011 (PMC12021413; doi:10.7554/eLife.101011)

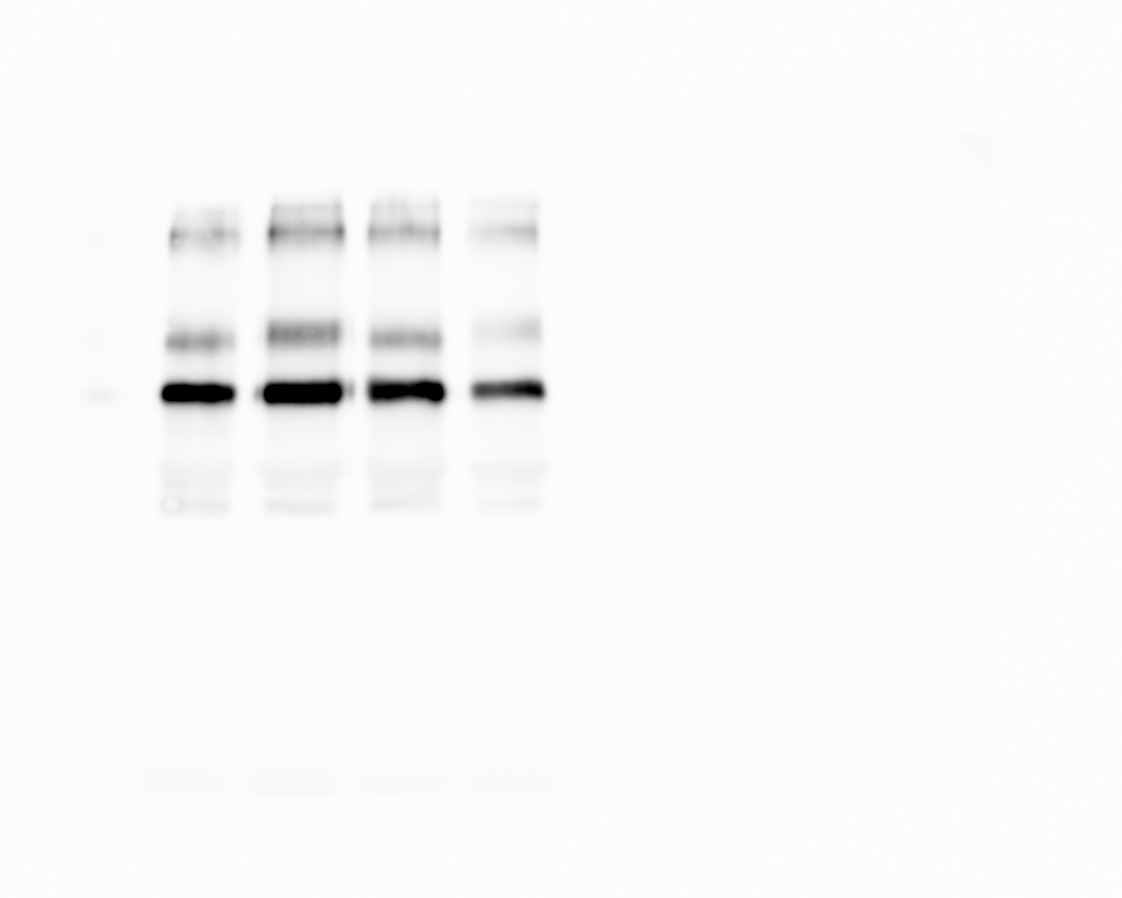

Supplement: Figure 3—figure supplement 1—source data 2. [file elife-101011-fig3-figsupp1-data2.zip › blots/photoclick cholesterol snap.tif]

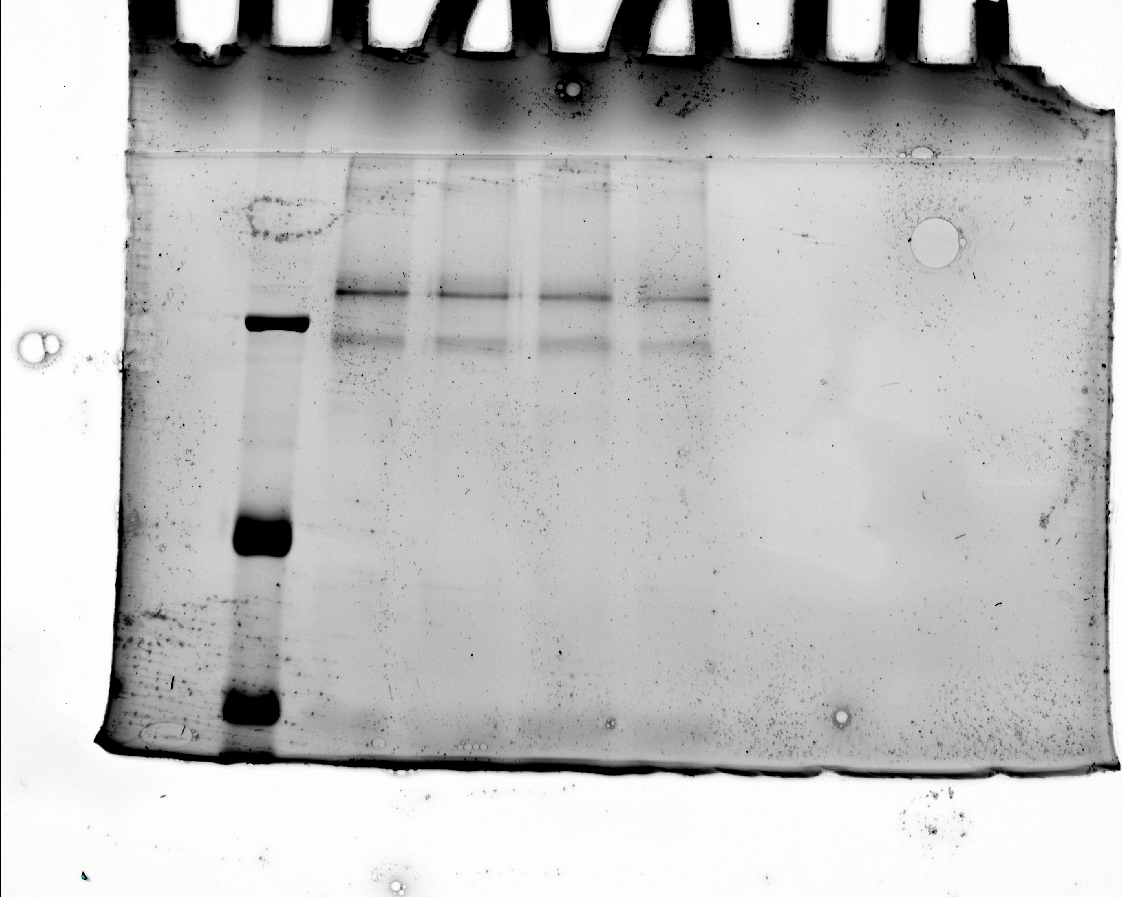

Supplement: Figure 3—figure supplement 1—source data 2. [file elife-101011-fig3-figsupp1-data2.zip › blots/photoclick cholesterol.tif]
